# A Modality Alignment and Fusion-Based Method for Around-the-Clock Remote Sensing Object Detection

**DOI:** 10.3390/s25164964

**Published:** 2025-08-11

**Authors:** Yongjun Qi, Shaohua Yang, Jiahao Chen, Meng Zhang, Jie Zhu, Xin Liu, Hongxing Zheng

**Affiliations:** 1 School of Computer Science and Engineering, North China Institute of Aerospace Engineering, Langfang 065000, China; qyj@nciae.edu.cn (Y.Q.); zhangmeng@stumail.nciae.edu.cn (M.Z.); 2 Hebei Collaborative Innovation Center of Micro Nano Satellites, North China Institute of Aerospace Engineering, Langfang 065000, China; ysh0130@stumail.nciae.edu.cn; 3 School of Information Engineering, Capital Normal University, Beijing 100048, China; 1231003001@cnu.edu.cn; 4 School of Optoelectronic Engineering, Xidian University, Xi’an 710071, China; xinliu@mail.xidian.edu.cn; 5 School of Electronics and Information Engineering, Hebei University of Technology, Tianjin 300401, China

**Keywords:** cross-modal remote sensing object detection, around-the-clock, adverse weather, modality differences

## Abstract

Cross-modal remote sensing object detection holds significant potential for around-the-clock applications. However, the modality differences between cross-modal data and the degradation of feature quality under adverse weather conditions limit detection performance. To address these challenges, this paper presents a novel cross-modal remote sensing object detection framework designed to overcome two critical challenges in around-the-clock applications: (1) significant modality disparities between visible light, infrared, and synthetic aperture radar data, and (2) severe feature degradation under adverse weather conditions including fog, and nighttime scenarios. Our primary contributions are as follows: First, we develop a multi-scale feature extraction module that employs a hierarchical convolutional architecture to capture both fine-grained details and contextual information, effectively compensating for missing or blurred features in degraded visible-light images. Second, we introduce an innovative feature interaction module that utilizes cross-attention mechanisms to establish long-range dependencies across modalities while dynamically suppressing noise interference through adaptive feature selection. Third, we propose a feature correction fusion module that performs spatial alignment of object boundaries and channel-wise optimization of global feature consistency, enabling robust fusion of complementary information from different modalities. The proposed framework is validated on visible light, infrared, and SAR modalities. Extensive experiments on three challenging datasets (LLVIP, OGSOD, and Drone Vehicle) demonstrate our framework’s superior performance, achieving state-of-the-art mean average precision scores of 66.3%, 58.6%, and 71.7%, respectively, representing significant improvements over existing methods in scenarios with modality differences or extreme weather conditions. The proposed solution not only advances the technical frontier of cross-modal object detection but also provides practical value for mission-critical applications such as 24/7 surveillance systems, military reconnaissance, and emergency response operations where reliable around-the-clock detection is essential.

## 1. Introduction

Remote sensing object detection has emerged as a fundamental technology in computer vision, with widespread applications spanning intelligent surveillance systems [1], wildlife conservation [2], and military reconnaissance operations [3]. The evolution of convolutional neural networks (CNNs) has significantly advanced detection methodologies [1,2,3,4], yet most existing approaches remain constrained to visible-light imagery or high-visibility conditions [5,6]. This limitation becomes particularly problematic when dealing with low-visibility scenarios (e.g., nighttime) or adverse weather conditions (e.g., haze, fog) [5,6], where single-modal detectors often fail to maintain robust performance. Consequently, researchers have increasingly turned to multi-modal solutions that combine visible light with complementary modalities such as infrared imagery or synthetic aperture radar (SAR) [7,8], aiming to achieve reliable around-the-clock detection through comprehensive feature fusion [9,10].

Prior work in cross-modal remote sensing object detection has primarily addressed two technical challenges through various approaches. For modality alignment, Sun et al. [11] proposed UA-CMDet to enhance spatial and semantic alignment through joint optimization, though its fusion strategy showed limited flexibility in complex scenarios. Song et al. [12] developed CMADet with cross-modal multi-scale attention, yet struggled with complete feature alignment in dynamic scenes. Regarding feature fusion, Yuan et al. [13] introduced C^2^Former using Transformer’s cross-attention mechanism, while Wang et al. [14] improved YOLO’s fusion strategy in YOLOFIV, both achieving notable but incomplete progress [13,14,15,16]. Other notable contributions include Qingyun et al.’s CFT framework [15] for unified feature space mapping and Bao et al.’s DDCI [16] with deep dual cross-interaction, though these methods still face limitations in noise suppression and dynamic scene adaptation [15,16]. Recent advances like GAFF [17], ProbEN [18], and CSSA [19] have further enriched the field through novel fusion architectures and probabilistic modeling, while knowledge distillation techniques (KD [20], DKD [21]) and local descriptor methods (LD [22], CoLD [23]) have provided alternative optimization pathways [20,21,22,23]. Attention mechanisms have also played a pivotal role, from SENet’s channel-wise modulation [24] to transformer-based global context modeling [25,26,27,28], particularly benefiting cross-modal interpretation tasks [25].

Despite these advancements, two critical limitations persist in current research:

(1) Existing methods often address either modality differences or feature degradation in isolation, failing to fully exploit spatial-semantic complementary information across visible light, infrared, and SAR modalities [29,30];
(2) Most solutions lack robust mechanisms for handling severe feature quality deterioration under extreme weather conditions, where visible light images suffer from illumination insufficiency while infrared images are affected by thermal noise [30]. These limitations become particularly apparent in mission-critical applications requiring operational capability, where even state-of-the-art methods like CCFINet [31] and CrossFormer [32] show compromised accuracy during modality transitions or weather extremes [31,32].

To overcome these challenges, this paper makes four key contributions:

(1) We propose a comprehensive cross-modal detection framework that simultaneously addresses modality differences and feature degradation through synergistic module design.
(2) We develop a multi-scale feature extraction module (MSFEM) with a multi-branch convolutional architecture that captures diverse receptive fields, effectively compensating for lost visible-light features in adverse conditions.
(3) We introduce a feature interaction module (FIM) employing cross-attention mechanisms to model long-range inter-modal dependencies while implementing dynamic feature selection for noise suppression.
(4) We design a feature correction fusion module (FCFM) that performs spatial boundary alignment and channel-wise consistency optimization through novel correction mechanisms.

**Figure 1:**
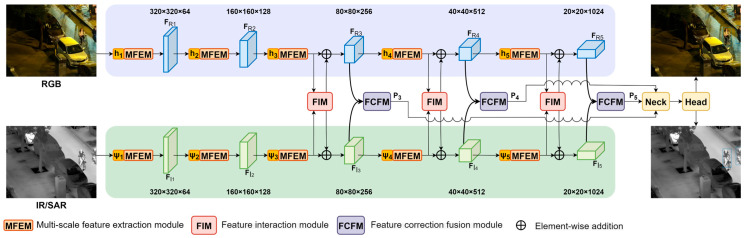
Architecture of the cross-modal remote sensing object detection network.

As illustrated in Figure 1, our complete architecture demonstrates superior performance across three challenging datasets (LLVIP, OGSOD, Drone Vehicle), achieving mAP improvements of 2.2–13.6% over existing methods while maintaining efficient computational characteristics, a critical advantage for real-time applications. The network has a dual-stream architecture, with primary modules including the multi-scale feature extraction module, the feature interaction module, and the feature correction fusion module. where $h_{i}$ and $\psi_{i}$ denote the convolutional modules for the RGB and IR modalities at the i-th layer, respectively. $F_{Ri}$ and $F_{Ii}$ represent the feature maps of the corresponding modalities at the i-th layer. $P_{3}$, $P_{4}$, and $P_{5}$ serve as the inputs to the feature pyramid of the last three stages.

The remainder of this paper is organized as follows: Section 2 details our methodology, including mathematical formulations and module designs. Section 3 presents experimental results and comparative analyses. Section 4 concludes with implications and future research directions.

## 2. Methodology

### 2.1. Problem Formulation and Network Overview

Building upon the challenges identified in Section 1, particularly the modality disparities between visible light (RGB), infrared (IR), and synthetic aperture radar (SAR) data, along with feature degradation under adverse weather conditions, we propose a dual-stream architecture with three specialized modules. As shown in Figure 1, our framework processes aligned image pairs through parallel feature extraction streams, followed by multi-scale enhancement and cross-modal fusion.

The complete processing pipeline can be formulated as follows:

(1) $$F_{out}=FCFM(FIM\left(MSFEM(F_{rgb})\right),\left(MSFEM(F_{ir})\right)),$$

where $F_{rgb}$ and $F_{ir}$ denote the input feature maps from RGB and IR/SAR modalities, respectively, and $F_{out}$ represents the final fused features for detection.

In designing this architecture, we aimed to create a coherent and comprehensive solution that addresses significant modality disparities and ensures robust performance under unfavorable lighting conditions. The multi-scale feature extraction module (MSFEM) captures diverse receptive fields using a multi-branch structure with convolutional operations of different scales, compensating for lost visible-light features. The feature interaction module (FIM) then employs cross-attention mechanisms to model long-range inter-modal dependencies, dynamically fusing features from different modalities to leverage their strengths while suppressing noise. Finally, the feature correction fusion module (FCFM) performs spatial boundary alignment and channel-wise consistency optimization, ensuring the accurate and consistent fusion of features. Together, these modules form a robust and reliable framework for cross-modal object detection, capable of handling multi-modal remote sensing data under various environmental conditions.

### 2.2. Multi-Scale Feature Extraction Module

**Figure 2:**
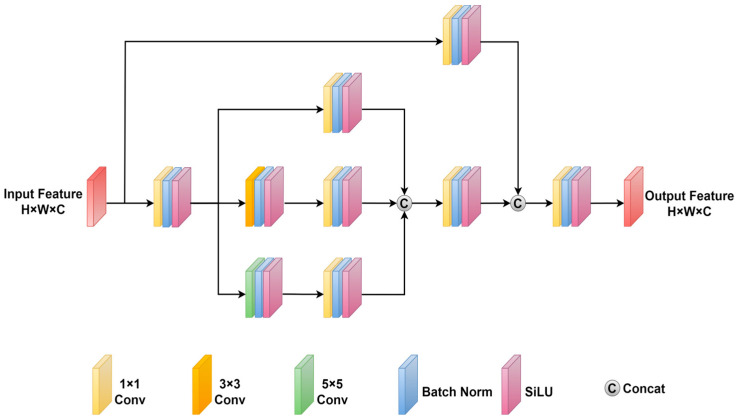
Multi-scale feature extraction module.

Remote sensing images typically exhibit a large number of small objects that are densely distributed. Traditional convolutional neural networks (CNNs), limited by their fixed receptive fields, struggle to capture rich object feature information. To address this issue and enhance the network’s perception of small objects, this paper proposes a multi-scale feature extraction module (MSFEM). By employing convolutional operations of different scales within a multi-branch structure, the multi-scale feature extraction module can extract object feature information from multiple receptive field sizes. This enables the network to better understand the relationships between pixels in the feature map, thereby improving detection accuracy. Figure 2 illustrates the detailed structure of the multi-scale feature extraction module. The multi-scale feature extraction module consists of four feature extraction branches. The main branch includes convolutional modules of 1 × 1, 3 × 3, and 5 × 5, while the residual branch contains only a 1 × 1 convolutional module. It is worth noting that each convolutional module comprises three components: a convolutional layer, batch normalization, and a SiLU activation function.

The feature extraction process of the multi-scale feature extraction module is described as follows. First, the input feature map (H × W × C) is divided into a residual branch and a main branch. In the main branch, a 1 × 1 convolutional operation is initially performed to maintain the size of the feature map. Subsequently, the main branch is further divided into three convolutional branches. The first convolutional branch employs a single 1 × 1 convolutional module for feature extraction. The second convolutional branch applies a 3 × 3 convolutional module followed by a 1 × 1 convolutional module. The third convolutional branch utilizes a 5 × 5 convolutional module followed by a 1 × 1 convolutional module. Next, the three convolutional branches are concatenated along the channel dimension and passed through a 1 × 1 convolutional module, resulting in an output feature map size of H × W × C. In the residual branch, a single 1 × 1 convolutional module is used, yielding an output feature map size of H × W × C. The output feature maps from the main branch and the residual branch are then concatenated along the channel dimension, producing a feature map size of H × W × 2C. To match the channel number of the original input feature map, a 1 × 1 convolutional module is employed for dimensionality reduction, resulting in the output feature map of the multi-scale feature extraction module. At this point, the output feature map of the multi-scale feature extraction module has the same size as the input feature map, namely H × W × C.

### 2.3. Feature Interaction Module

**Figure 3:**
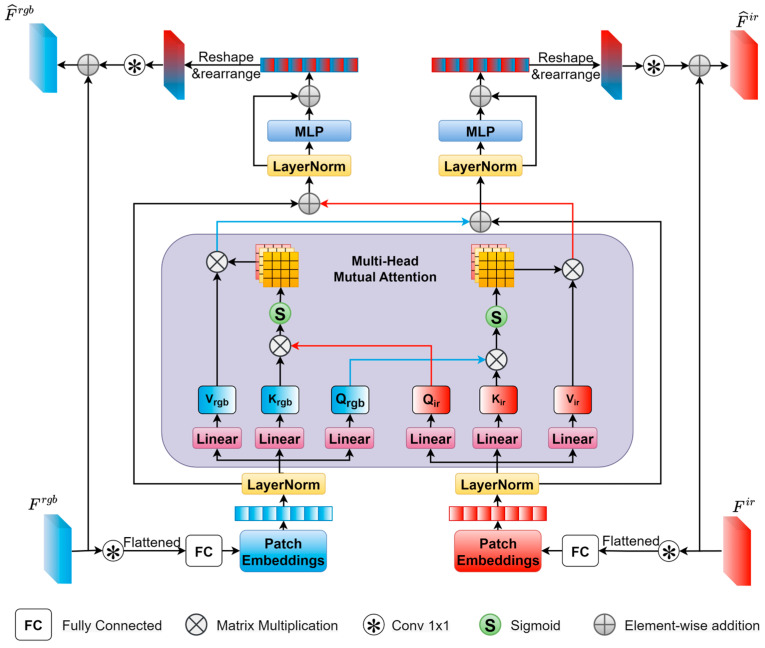
Feature interaction module.

To fully exploit the complementarity between different modalities and capture the long-range global dependencies between modalities, this paper proposes a feature interaction module. As shown in Figure 3, the feature interaction module takes $F_{rgb}$ and $F_{ir}$ as inputs and outputs the corresponding enhanced features ${\hat{F}}^{rgb}$ and ${\hat{F}}^{ir}$ after feature interaction.

Generally, the input features of the feature interaction module are first converted into patch embedding sequences and then fed into the cross-attention sub-module to perform cross-modal interactions by modeling the long-range relationships between the two modalities. Subsequently, the fused patch embeddings are, respectively, input into two feed-forward networks, and then reshaped and rearranged to obtain the interacted features. Finally, the enhanced features are obtained by the element-wise addition of the interacted features and the input features.

Initially, two 1 × 1 convolutional layers are employed to reduce the channel number of $F_{rgb},F_{ir}\in R^{H\times W\times C}$ resulting in $F_{rgb}^{\prime },F_{ir}^{\prime }\in R^{H\times W\times C\prime }$, where (H, W) represents the spatial resolution of the feature maps, C is the original channel number, and C′ is the reduced channel number. Subsequently, the multi-head cross-attention and the two feed-forward networks require sequences as inputs. Therefore, $F_{rgb}^{\prime }$ and $F_{ir}^{\prime }$ are reshaped into two flattened 2D patch sequences $x^{rgb},x^{ir}\in R^{N\times d_{patch}}$, where $d_{patch}=P^{2}\times C\prime$ is the dimension of each flattened patch, with a resolution of (P, P), and $N=HW/P^{2}$ is the number of patches. Thereafter, through two fully connected layers, $x_{rgb}$ and $x_{ir}$ are encoded into patch embeddings $x_{rgb}^{emb},x_{ir}^{emb}\in R^{N\times d}$, where d is the dimension of each patch embedding. Finally, $x_{rgb}^{emb},x_{ir}^{emb}$ are further processed through two normalization layers.

As shown in Figure 3, the patch embeddings are fed into the multi-head cross-attention sub-module. Specifically, $x_{rgb}^{emb},x_{ir}^{emb}$ are linearly projected to generate their respective queries, keys, and values, denoted as $Q_{rgb},K_{rgb},V_{rgb}\in R^{N\times d}$ and $Q_{ir},K_{ir},V_{ir}\in R^{N\times d}$. Subsequently, scaled dot-product attention is applied to each head, which can be expressed as follows:

(2) $${head}_{rgb,i}=Softmax\left(Q_{rgb,i},\;\;\frac{K_{ir,i}}{\sqrt{d_{k}}}\right)V_{ir,i},$$

(3) $${head}_{ir,i}=Softmax(Q_{ir,i},\;\frac{K_{rgb,i}}{\sqrt{d_{k}}})V_{rgb,i},$$

where ${head}_{rgb,i}$ and ${head}_{ir,i}$ represent the i-th head of RGB and IR, respectively. The output of each head is concatenated, and then fed into a series of operations including dropouts, residual connections, normalization, and feed-forward. The final output is the interacted patch embeddings ${\dot{x}}_{rgb}^{emb},{\dot{x}}_{ir}^{emb}\in R^{N\times d}$.

Subsequently, two fully connected layers are employed to decode ${\dot{x}}_{rgb}^{emb},{\dot{x}}_{ir}^{emb}$ back into two interacted flattened patch sequences ${\dot{x}}_{rgb},{\dot{x}}_{ir}\in R^{N\times d_{patch}}$ These sequences are then reshaped and rearranged to obtain the interacted features with $C^{\prime }$ channels. Finally, two 1 × 1 convolutional layers are used to restore the channel number, generating the interacted features ${\dot{F}}_{rgb},{\dot{F}}_{ir}\in R^{H\times W\times C}$.

Finally, the interacted features are element-wise added to the original input features to obtain the enhanced features, which can be expressed as follows:

(4) $${\hat{F}}_{rgb}=F_{rgb}+{\dot{F}}_{rgb},$$

(5) $${\hat{F}}_{ir}=F_{ir}+{\dot{F}}_{ir},$$

### 2.4. Feature Correction Fusion Module

**Figure 4:**
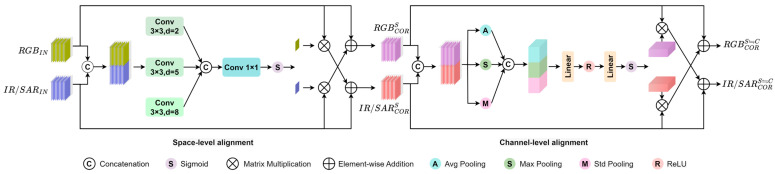
Feature correction fusion module.

The detailed information of the proposed feature correction fusion module is shown in Figure 4. It adaptively corrects and filters the complementary information between different modalities through spatial and channel correlations, achieving better cross-modal feature extraction and fusion. The order of feature correction affects the accuracy of the proposed model, as shown in Section 3.6. In this section, the specific structure is introduced in the order of spatial correction followed by channel correction.

(a) **Spatial correction first processes concatenated features:**

The spatial feature correction module involves aligning features from different modalities to enhance detection accuracy, as shown in Figure 4. Let the visible light image be denoted as ${RGB}_{IN}$, and the corresponding infrared image is denoted as ${IR}_{IN}$, where infrared can be replaced by other complementary modalities such as SAR. First, the features from different modalities are concatenated. Subsequently, an MLP layer is employed to perform layer-wise nonlinear transformations and feature learning on the concatenated features, resulting in the feature map $F^{S}\in R^{H\times W\times 2C}$. The MLP layer consists of three dilated convolutions, ReLU activation functions, and a 1 × 1 convolution layer, as shown in Equation (7). Next, the Sigmoid function is used to change the output range of $F^{S}$ to [0, 1], and it is further divided into two spatial weight maps, $\omega_{rgb}\in R^{H\times W\times C}$ and $\omega_{ir}\in R^{H\times W\times C}$, as shown in Equation (8).

(6) $$F^{S}=MLP(2C,C)(Comcat({RGB}_{IN},{IR}_{IN})),$$

(7) $$MLP=\sigma (Conv(Concat(rate2\left(Y^{C}\right),rate5\left(Y^{C}\right),rate8\left(Y^{C}\right)))),$$

(8) $$\omega_{rgb}^{S},\omega_{ir}^{S}=Spilt(\sigma (F^{S})),$$

where rate2, rate5, and rate8 denote the dilated convolution operations with dilation rates of 2, 5, and 8, respectively; σ represents the Sigmoid function.

By multiplying the obtained spatial correction feature map $\omega_{ir}$ with the corresponding input ${IR}_{IN}$, we achieve the spatial correction of the input ${RGB}_{IN}$. Subsequently, this corrected feature is added to ${RGB}_{IN}$ to obtain the spatially corrected feature map ${RGB}_{COR}^{S}$. Furthermore, a trainable parameter α(0≤α≤1) is introduced to control the dynamic weights of spatial and channel corrections. During the model training process, this parameter automatically adjusts based on the contributions of the two correction offsets to detection accuracy, thereby dynamically learning the optimal fusion ratio of the two sets of correction features. This enhances the model’s adaptability and robustness. The correction process can be represented by the following equation:

(9) $${RGB}_{COR}^{S}={RGB}_{IN}+{\alpha \omega }_{ir}^{S}\cdot {IR}_{IN},$$

(10) $${IR}_{COR}^{S}={IR}_{IN}+{\alpha \omega }_{rgb}^{S}\cdot {RGB}_{IN},$$

where ${RGB}_{COR}^{S}$ and ${IR}_{COR}^{S}$ represent the visible light and infrared feature maps that have undergone spatial correction, respectively; $\omega_{rgb}^{S}$ and $\omega_{ir}^{S}$ denote the spatial correction offset weights; and α signifies a learnable parameter during the spatial correction phase.

(b) **Channel correction then performs global optimization:**

Channel-level feature correction is utilized to further correct global information after spatial-level feature correction has been applied to the local information, in order to optimize the overall consistency of the features. The features ${RGB}_{COR}^{S}$ and ${IR}_{COR}^{S}$, which have undergone spatial-level feature correction, are first concatenated to obtain $X^{C}\in R^{H\times W\times 2C}$. For complex cross-modal features, a comprehensive pooling strategy is employed, including global average pooling to capture overall average information, global max pooling to capture globally salient features, and global standard deviation pooling to focus on feature variations and distribution information. This combination of various pooling operations can provide a more comprehensive and rich feature representation, enabling the model to adapt to different types of data and distributions. Subsequently, the global feature vectors obtained from these three pooling operations are concatenated to form a feature map $Y^{C}$ of size $R^{6C}$. Next, an MLP layer is applied to obtain $F^{C}\in R^{2C}$, and a Sigmoid activation function and slicing operation are used to obtain channel weight maps $\omega_{rgb}^{C}\in R^{C}$ and $\omega_{ir}^{C}\in R^{C}$ that contain global feature information. The aforementioned process can be represented as follows:

(11) $$X^{C}=Concat({RGB}_{COR}^{S},{IR}_{COR}^{S}),$$

(12) $$Y^{C}=Concat(Avg(X^{C}),Max(X^{C}),Std(X^{C})),$$

(13) $$F^{C}=MLP(6C,2C)(Y^{C}),$$

(14) $$MLP=Linear(C_{mid},C_{out})(ReLU(Linear(C_{in},C_{out})(\cdot ))),$$

(15) $$\omega_{rgb}^{C},\omega_{ir}^{C}=Spilt(\sigma (F^{C})),$$

where $Linear(C_{in},C_{out})(\cdot )$ represents a linear layer with input channels $C_{in}$ and output channels $C_{out},$ ReLU(·) denotes the ReLU activation function, and σ(·) represents the Sigmoid function.

In Equation (14), the MLP is composed of two linear layers and a rectifying linear unit; Avg(·), Max(·), and Std(·) represent global average pooling, global max pooling, and global standard deviation pooling, respectively; and σ denotes the Sigmoid function.

Similarly to spatial correction, channel feature correction can be represented in the following form:

(16) $${RGB}_{COR}^{S⇌C}={RGB}_{COR}^{S}+(1-\alpha )\cdot \omega_{IR}^{C}\cdot {IR}_{COR}^{S},$$

(17) $${IR}_{COR}^{S⇌C}={IR}_{COR}^{S}+(1-\alpha )\cdot \omega_{RGB}^{C}\cdot {RGB}_{COR}^{S},$$

where ${RGB}_{COR}^{S}$ and ${IR}_{COR}^{S}$ denote the visible light and infrared feature maps that have been spatially corrected, respectively. The terms ${RGB}_{COR}^{S⇌C}$ and ${IR}_{COR}^{S⇌C}$ refer to the feature maps of visible light and infrared that have undergone both spatial and channel corrections. The parameter 1−α represents a learnable parameter in the channel correction phase, where α in Equation (16) is the same as in Equation (9), governing the relative significance of the spatial and channel correction phases. The parameters $\omega_{IR}^{C}$ and $\omega_{RGB}^{C}$ signify the weights assigned to the channel correction offsets for the infrared and visible light modalities, respectively.

Finally, the features that have undergone both spatial and channel corrections are added element-wise to obtain the ultimately corrected and fused features, which can be represented as follows:

(18) $$Output={RGB}_{COR}^{S⇌C}+{IR}_{COR}^{S⇌C},$$

## 3. Experiments

### 3.1. Datasets

All experiments were evaluated on three datasets: LLVIP [33], OGSOD, and Drone Vehicle.

(a) **LLVIP.** The LLVIP dataset is a highly challenging multi-spectral dataset for pedestrian detection under low-light conditions. Collected in low-light environments, this dataset makes accurate pedestrian detection in the RGB modality extremely challenging. The dataset comprises 12,025 pairs of aligned RGB-T images in the training set and 3463 pairs of images in the validation set, with each image having a resolution of 1024 × 1280.
(b) **OGSOD.** The OGSOD dataset is a recently released optical-SAR paired dataset for cross-modal remote sensing object detection. It includes a training set with 14,665 image pairs and a test set with 3666 image pairs, containing over 48,000 instance annotations in total. All images have a size of 256 × 256. Three categories are annotated, including bridges, tanks, and ports.
(c) **Drone Vehicle.** The Drone Vehicle dataset is a large-scale aerial optical-infrared dataset captured by drones, containing 28,439 image pairs and 953,087 vehicle annotation instances. This dataset covers a variety of scenes, including urban roads, residential areas, parking lots, and varying lighting conditions from day to night. The dataset is divided into five vehicle categories (cars, buses, trucks, vans, and cargo vehicles) and provides rich oriented bounding box annotations. To facilitate processing, the white borders of the images were removed, and the image size was uniformly adjusted to 640 × 512.

### 3.2. Implementation Details

This paper employs the Stochastic Gradient Descent (SGD) optimizer with an initial learning rate of 1 × 10^−2^, a momentum of 0.937, and a weight decay of 0.0005. A cosine annealing learning rate scheduler is applied, reducing the learning rate to 1 × 10^−6^ over 200 epochs. All models were trained for 200 epochs on a single NVIDIA RTX 3090 GPU with a batch size of 16. To achieve better performance, the YOLO model pre-trained on the COCO dataset [34] was utilized for weight initialization. Regarding data augmentation, the mosaic method, which combines four training images into one, was employed.

### 3.3. Evaluation Metrics

All models were evaluated using three object detection metrics introduced by MS-COCO: mean Average Precision (mAP), mAP at IoU = 0.5 (mAP50), and mAP at IoU = 0.75 (mAP75).

(19) $$mAP=\frac{1}{n}\sum_{i=0}^{n}{AP}_{i}=\frac{1}{n}\sum_{i=0}^{n}\int_{0}^{1}P_{i}(r)dr,$$

where

(20) $$\begin{array}{ll} {AP}_{i} & =\int_{0}^{1}P_{i}\left(r\right)dr\\ & =\int_{0}^{1}Precision\;d\left(Recall\right)\\ & =\int_{0}^{1}\frac{TP}{TP+FP}d\frac{TP}{TP+FN} \end{array}$$

TP means true positive, which is when a predicted box by detectors and the ground truth (GT) meet the intersection over union (IoU) threshold; otherwise, it will be considered as a false positive (FP). False negative (FN) means there is a true object, but the detector does not find it. Equation (20) indicates that AP is the integral of the Precision–Recall Curve (PRC) for each category. mAP50 computes the mean of all the AP values for all categories at IoU = 0.50 in Equation (19). Similarly, mAP75 calculates the mean at IoU = 0.75. mAP is the primary challenge metric, which can be formulated as the mean at IoU = 0.50:0.05:0.95. Obviously, it is much stricter than the other two metrics.

### 3.4. Comparison with Existing Methods

To validate the performance advantages of the proposed cross-modal remote sensing object detection model under around-the-clock conditions and in complex scenarios, this section conducts comparative experiments on the LLVIP, OGSOD, and Drone Vehicle datasets against uni-modal and state-of-the-art cross-modal methods. These datasets cover a variety of scenes including traffic cameras, satellite images, and drone perspectives, ensuring the comprehensiveness and objectivity of the model evaluation due to their diverse viewpoints and environmental conditions. Overall, our method achieves significant improvements in mAP compared to both uni-modal methods and existing cross-modal approaches, with specific gains of 4.4% on LLVIP (66.3% vs. 61.9%), 12.3% on OGSOD (58.6% vs. 46.3%), and 8.3% on Drone Vehicle (71.7% vs. 63.4%).

On the LLVIP dataset, as shown in Table 1, the proposed cross-modal remote sensing object detection model demonstrates significant performance improvements compared to both uni-modal object detection models and existing cross-modal approaches. Specifically, compared to uni-modal methods, the cross-modal remote sensing object detection model achieved an improvement in mAP50 ranging from 2.7% to 12%. When compared to advanced methods such as CCFINet and CFT, the cross-modal remote sensing object detection model surpassed them with an mAP50 of 97.9% (compared to 97.6% and 97.5% for CCFINet and CFT, respectively), and achieved an overall mAP of 66.3%.

On the OGSOD dataset, as illustrated in Table 2, the cross-modal remote sensing object detection model further solidifies its leading position in cross-modal object detection. Under the RGB+SAR modality, the model achieved an mAP50 of 94.5% and an overall mAP of 58.6%, surpassing advanced methods such as CoLD and GI Imitation, which achieved mAP50 scores of 87.6% and 87.1%, respectively.

On the Drone Vehicle dataset, as demonstrated in Table 3, the proposed method was evaluated and compared with the state-of-the-art approaches. The model exhibited notable performance improvements under conditions with smaller objects and more complex environments. Specifically, compared to uni-modal methods, the cross-modal remote sensing object detection model improved the mAP50 by 15.6% to 32.18%. When compared to advanced methods such as COMO and CFT, the cross-modal remote sensing object detection model outperformed them with an mAP50 of 86.3% (compared to 86.1% and 84.3% for COMO and CFT, respectively), and achieved an overall mAP of 71.7%.

We compare the model size, computational complexity, and inference speed of our method against baselines on the Drone Vehicle dataset, as shown in Table 4. The metrics include parameter count (Params, M), FLOPs (@640, G), and FPS (Hz), measured on a single NVIDIA RTX 3090 GPU. Our model achieves low FLOPs of 14.36 G, the lowest among compared methods (e.g., 18.45 G for DaFF, 176.00 G for GM-DETR), and a high FPS of 226.2 Hz, surpassing most baselines (e.g., 217.4 Hz for ICAFusion, 208.3 Hz for CMADet). While our parameter count (68.43 M) is higher than several baselines (e.g., 4.83 M for SuperYOLO, 20.15 M for ICAFusion), the additional parameters in MSFEM’s multi-branch convolutions (3 × 3, 5 × 5, 7 × 7), FIM’s eight attention heads, and FCFM’s fusion layers contribute to a significant mAP improvement (e.g., 71.7% vs. 63.4% for the dual-stream baseline on Drone Vehicle).

### 3.5. Visualization Results

**Figure 5:**
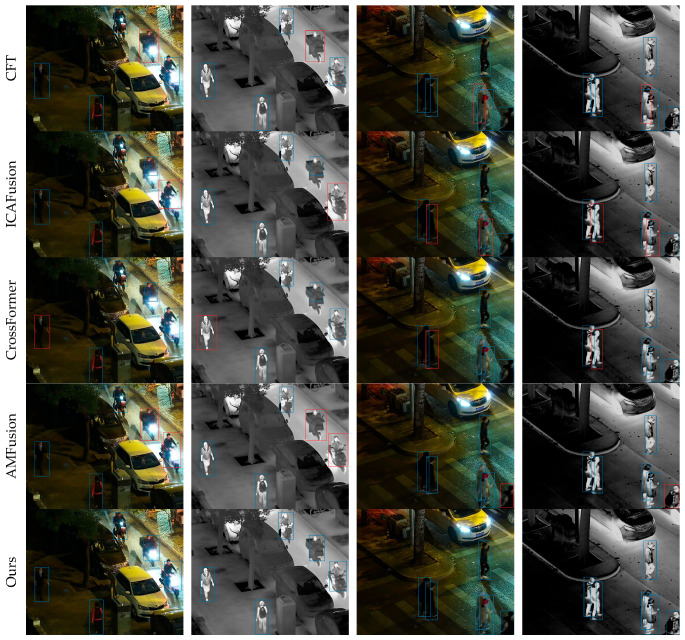
Visualization of detection results on LLVIP. We compare cross-modal remote object detection with four other methods. Blue bounding boxes denote TPs; red bounding boxes denote FNs.

**Figure 6:**
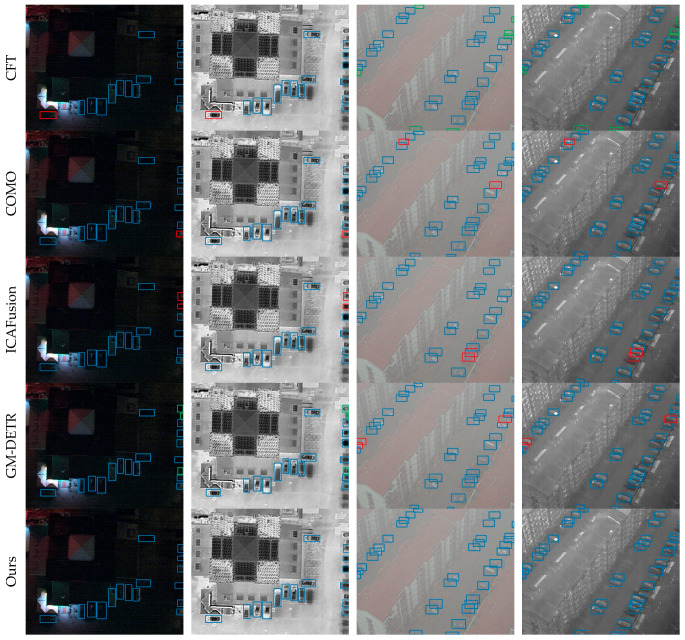
Visualization of detection results on Drone Vehicle. We compare cross-modal remote object detection with four other methods. Blue bounding boxes denote TPs; red bounding boxes denote FNs; red bounding boxes denote FPs.

**Figure 7:**
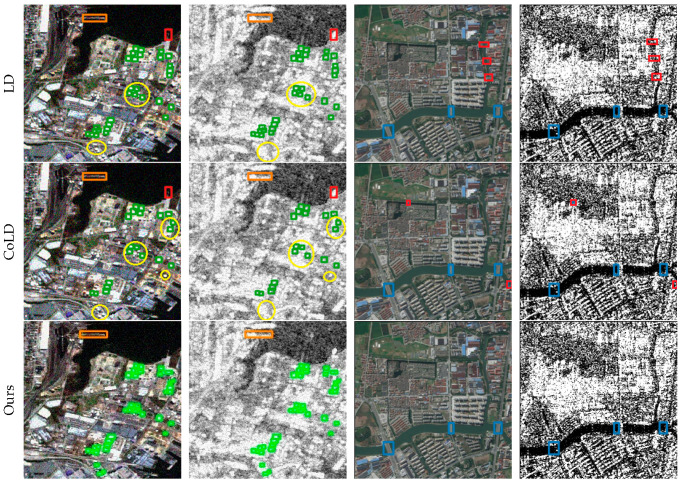
Visualization of detection results on Drone Vehicle. We compare cross-modal remote object detection with four other methods. Green, orange and blue bounding boxes denote TPs; red bounding boxes denote FNs; yellow bounding boxes denote FPs.

**Figure 8:**
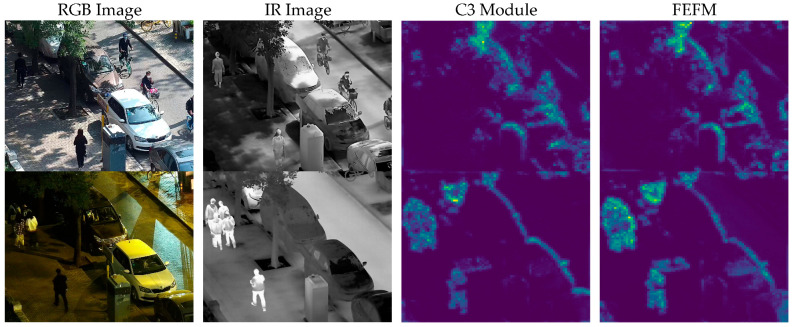
A comparison of feature map visualizations between the C3 (CSP Bottleneck with 3 convolutions) module and the multi-scale feature extraction module, with RGB and IR scenes divided into day and night. The third column displays the visualization results of the C3 module, while the fourth column shows the visualization results of the multi-scale feature extraction module.

**Figure 9:**
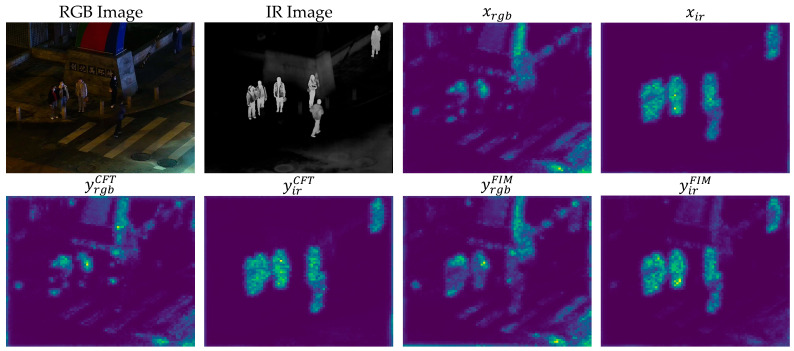
The visualization results of the feature interaction module. Where $x_{rgb}$ and $x_{ir}$ represent the visualization results of visible light and infrared images, respectively; $y_{rgb}^{CFT}$ and $y_{ir}^{CFT}$ denote the complementary feature visualization results of infrared and visible light by the CFT module; $y_{rgb}^{FIM}$ and $y_{ir}^{FIM}$ indicate the complementary feature visualization results of infrared and visible light by the feature interaction module.

**Figure 10:**
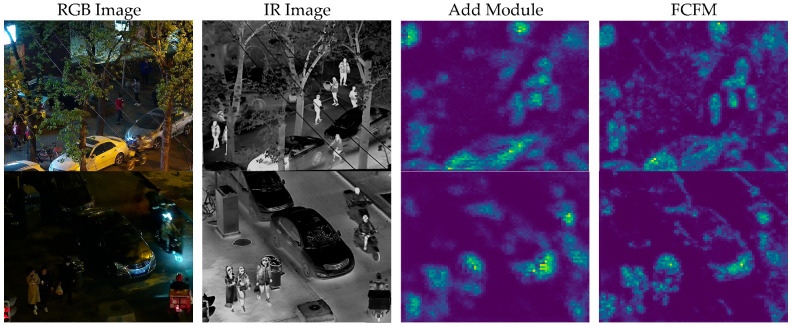
Presents the comparative feature map visualization results between element-wise addition and the feature correction fusion module. The first and second columns represent the infrared and visible light images of night scenes, respectively; the third column shows the visualization results of the feature maps from Add (element-wise addition); the fourth column displays the visualization results of the feature maps from the feature correction fusion module.

In addition to the comparisons with existing methods, this paper also presents the detection results of the cross-modal remote sensing object detection model on the three datasets in Figure 5, Figure 6 and Figure 7 to visually demonstrate the effectiveness of the proposed method. These figures illustrate the model’s ability to accurately detect objects under various conditions, highlighting its robustness and the advantages of integrating information from multiple modalities.

(a). Detection results on the LLVIP dataset: Figure 5 shows the detection outcomes for LLVIP, with rows from top to bottom representing CFT, ICAFusion, CrossFormer, AMFusion, and the cross-modal remote sensing object detection network. It is evident that the first three methods exhibit various degrees of missed detections due to insufficient extraction of complementary features. In contrast, the cross-modal remote sensing object detection network significantly improves the detection results. This confirms the effectiveness of the method proposed in this paper for cross-modal object detection images.

(b). Detection results on the Drone Vehicle dataset: Figure 6 displays the performance of the proposed cross-modal remote sensing object detection model alongside other methods, arranged from top to bottom as CFT, COMO, ICAFusion, GM-DETR, and the cross-modal remote sensing object detection network. Due to the impact of lighting and extreme weather conditions, several methods fail to fully exploit the advantages between infrared and visible light, leading to varying degrees of missed and false detections. In contrast, this paper’s method enhances the interaction between visible light and infrared features, effectively integrating complementary information, significantly reducing missed and false detections, and thus outperforming the comparative methods. This also demonstrates the effectiveness of the approach proposed in this paper.

(c). Detection results on the OGSOD dataset: Figure 7 illustrates the visual detection performance of several methods on the OGSOD dataset. From top to bottom, these are LD, CoLD, and the cross-modal remote sensing object detection method. Due to the noise and scattering effects in SAR images, there is inconsistency and redundancy between modalities, leading to varying degrees of missed and false detections in the results of other methods. The feature interaction module and the feature correction fusion module proposed in this paper aim to reduce modal inconsistency and redundancy, enabling the network to achieve better classification and more true positives (TPs).

(d). Visualization results of the multi-scale feature extraction module: As shown in Figure 8, this paper demonstrates the performance of the Multi-Scale Feature Extraction Module (MSFEM) in cross-modal remote sensing object detection through feature map visualization. To verify the effectiveness of this module, feature maps were visualized in Figure 8 and compared with the performance of the C3 module. The third column of Figure 8 shows the feature maps of the C3 module, indicating that it fails to adequately extract the edges and texture features of the objects, especially in multi-scale object scenarios. In contrast, the MSFEM accurately extracts key features and clearly delineates the contours of the objects through multi-scale feature fusion, particularly under conditions of low visibility or high noise, demonstrating its effectiveness in cross-modal object detection. Under daylight conditions, the MSFEM shows a significant ability to capture detailed features and textures, which is crucial for object detection in well-lit environments. The feature maps generated by the MSFEM exhibit higher clarity and better-defined object boundaries compared to those from the C3 module, which often struggles with noise and less defined edges. Under low-light (nighttime) conditions, the MSFEM’s multi-scale feature fusion is particularly effective. It compensates for the loss of visible-light features by leveraging information from multiple scales, thereby enhancing the overall feature quality. The feature maps from the MSFEM under low-light conditions show better contrast and less noise, which is essential for accurate object detection in such challenging environments.

(e). Visualization results of the feature interaction module: As shown in Figure 9, this paper demonstrates the performance of the feature interaction module in cross-modal remote sensing object detection through feature map visualization. To verify the effectiveness of this module, feature maps were visualized in Figure 9 and compared with the performance of the CFT module. Where $x_{rgb}$ and $x_{ir}$ represent the feature maps of visible light and infrared images, respectively; $y_{rgb}^{CFT}$ and $y_{ir}^{CFT}$ denote the complementary feature maps by the CFT module; $y_{rgb}^{FIM}$ and $y_{ir}^{FIM}$ indicate the complementary feature maps by the feature interaction module. From Figure 9, it can be observed that while the CFT module ($y_{rgb}^{CFT}$ and $y_{ir}^{CFT}$) can extract basic complementary features between visible light and infrared light, it has limitations when modeling long-distance dependencies between modalities. Specifically, the feature maps of the CFT module lack clarity in distinguishing object edges from background noise, leading to insufficient precision in complementary feature extraction. Additionally, in complex scenarios, the CFT module is susceptible to noise and background interference, further reducing the efficiency and accuracy of feature extraction. In contrast, this module effectively models the long-distance relationships between visible light and infrared modalities by converting features into patch-embedded sequences and utilizing a cross-attention mechanism, significantly enhancing the quality of feature maps. The cross-attention mechanism dynamically captures complementary information between visible light and infrared, enhancing the clarity of feature maps at object edges and contours. Furthermore, the module optimizes the semantic information of feature maps through a feed-forward network, further highlighting object features. Through the synergistic effect of these mechanisms, the module effectively extracts complementary features between visible light and infrared light, suppresses background noise and modal redundancy, and significantly improves the efficiency and accuracy of feature extraction.

(f). Visualization results of the feature correction fusion module: To verify the effectiveness of the feature correction fusion module (FCFM), this paper visualizes the feature maps in Figure 10 and compares its performance with the element-wise addition method. Figure 10 shows a comparison of feature maps between the element-wise addition and the FCFM. The first row displays the visible light and infrared images under well-lit night conditions, while the second row shows the same under low-light (extremely dark) conditions. The third column presents the feature maps from element-wise addition, and the fourth column shows the feature maps from the FCFM. Under well-lit night conditions, the feature maps from element-wise addition can extract basic edges and texture features but struggle with spatial inconsistencies and feature redundancies between visible light and infrared modalities. This leads to less clarity in distinguishing object edges from background noise, resulting in insufficient feature extraction accuracy. In contrast, the FCFM significantly improves the quality of feature maps through spatial and channel-level feature correction mechanisms. Spatial feature correction aligns visible light and infrared features, effectively eliminating local spatial differences and enhancing the clarity of object features. Channel-level feature correction optimizes the semantic information of feature maps through global optimization, further highlighting object features. These mechanisms work synergistically to enhance feature extraction accuracy and robustness under well-lit night conditions. Under low-light (extremely dark) conditions, the element-wise addition method is particularly prone to noise and background interference in complex night scenes, further reducing detection accuracy. The feature maps from element-wise addition lack clarity and are often overwhelmed by background noise. The FCFM, however, demonstrates significant improvements in feature map quality under extremely low-visibility conditions. Spatial feature correction effectively aligns visible light and infrared features, reducing local spatial differences and enhancing object feature clarity. Channel-level feature correction optimizes the semantic information of feature maps through global optimization, further highlighting object features. Through these mechanisms, the FCFM effectively suppresses background noise and modal redundancy, significantly improving detection accuracy in complex night scenes.

### 3.6. Ablation Study

(1) In this section, various ablation studies were conducted to examine the effects of the multi-scale feature extraction module, the feature interaction module, and the feature correction fusion module on the LLVIP, OGSOD, and Drone Vehicle datasets. In Table 5, the detection performance on different datasets (LLVIP, OGSOD, and Drone Vehicle) is compared. The best records and improvements are indicated by bold and upward arrows (⬆), respectively. On the LLVIP dataset, the mAP values for RGB-only and IR-only are 50.0% and 61.9%, respectively, with IR-only detection results surpassing RGB-only, a trend that is also observed in the Drone Vehicle dataset. However, on the OGSOD dataset, the performance of RGB-only is slightly better than that of SAR-only (mAP: 6.1%⬆). This may be due to the presence of numerous low-light scenarios in LLVIP and Drone Vehicle, leading to the loss of effective object areas, whereas the OGSOD dataset contains relatively more SAR noise and lacks features that are as easily recognizable and identifiable as those in RGB and IR. Furthermore, comparing the mAP of IR-only and dual-stream baselines, the simple dual-stream network fails to fully exploit the inherent complementarity between different modalities. Moreover, these rudimentary approaches may increase the difficulty of network learning and exacerbate the imbalance between modalities, leading to a decrease in performance. From Table 5, it can be seen that after adopting the proposed MSFEM (multi-scale feature extraction module), FIM (feature interaction module), and FCFM (feature correction fusion module), the detection model’s performance has improved on all three datasets. Particularly for the OGSOD dataset, the evaluation metric mAP75 increased by 23.99%, and the mAP improved by 12.3%.

Table 5 demonstrates that the MSFEM enhances modality alignment and feature compensation, improving mAP by up to 4.9% (Drone Vehicle). The FIM addresses noise suppression and inter-modal dependencies, boosting mAP75 by 3.2% on OGSOD. The FCFM improves dynamic scene adaptation, with a 2.0% mAP gain on Drone Vehicle. These results validate our framework’s ability to address the limitations of prior work in modality alignment, feature degradation, and dynamic scenes.

(2) To further explore the impact of the feature correction fusion module on the cross-modal remote sensing object detection model, this paper conducted ablation experiments on the order of spatial and channel dimension feature corrections. “Channel → Spatial” indicates that channel dimension correction is performed first, followed by spatial dimension correction, while “Spatial → Channel” is the reverse order. As shown in Table 6, compared to performing channel dimension feature correction first, performing spatial feature correction first can increase the mAP by 2.8%. The paper suggests that this is because the intuitive local appearance differences between different modalities are more significant than the abstract global semantic differences. Therefore, to achieve the best experimental results, it is recommended to first eliminate the larger local spatial differences through spatial dimension correction and then eliminate the smaller global semantic differences through channel dimension correction.

## 4. Conclusions

This paper has presented a comprehensive solution to the critical challenges in cross-modal remote sensing object detection, specifically addressing (1) modality disparities between visible light, infrared, and SAR data, and (2) feature degradation under adverse weather conditions. Our framework’s three core innovations—the multi-scale feature extraction module (MSFEM) for robust feature representation, the feature interaction module (FIM) for cross-modal alignment, and the feature correction fusion module (FCFM) for dynamic feature optimization—collectively achieve state-of-the-art performance across multiple benchmark datasets. Experimental results demonstrate significant improvements, including a 4.4% increase in mAP on the LLVIP dataset (66.3% vs. 61.9% for IR-only baselines), a 12.3% higher mAP on OGSOD (58.6%), and a 71.7% mAP on the challenging Drone Vehicle dataset, validating the framework’s effectiveness in handling both modality differences and weather-induced feature deterioration. The proposed architecture’s modular design offers practical advantages for real-world deployment in 24/7 surveillance systems, military reconnaissance, and emergency response scenarios where reliability under varying conditions is paramount.

Despite these advancements, three limitations suggest promising directions for future research: (1) Computational efficiency—while our model achieves real-time performance on standard GPUs, further optimization through lightweight architectures or neural architecture search could enable deployment on edge devices with strict power constraints. (2) Extreme small object detection—performance on sub-10-pixel objects in dense scenes (common in satellite imagery) could be enhanced through dedicated high-resolution feature preservation techniques. (3) Generalized multi-modal learning—extending the framework to incorporate additional modalities like LiDAR or hyperspectral data while maintaining parameter efficiency requires novel fusion strategies. Potential solutions include developing attention-based modality gating mechanisms or investigating dynamic network architectures that adaptively activate relevant modality pathways. These improvements would further solidify the framework’s position as a versatile solution for mission-critical around-the-clock detection systems. Additionally, future work will further explore the robustness of the model, particularly in practical RGB-IR/SAR misregistration scenarios (e.g., ±1–10 pixel shifts, small rotations, and scale jitter) and partial modality dropout (i.e., the availability of only a single modality at test time). Additionally, we will analyze the performance of the model as a function of the level of perturbation and provide qualitative examples. These investigations will help to better understand the adaptability and stability of the model under different conditions. Meanwhile, we plan to combine three modalities (e.g., visible light, infrared, and SAR) to further demonstrate the robustness and versatility of our method.

## Figures and Tables

**Table 1:** Comparison of performances on LLVIP datasets measured. Bold red indicates the best; bold blue indicates the second best.

Modality	Method	mAP50	mAP75	mAP
IR	Faster R-CNN	92.6	48.8	50.7
	SSD [35]	90.2	57.9	53.5
	YOLOv3	89.7	53.4	52.8
	YOLOv5 [36]	94.6	72.2	61.9
	YOLOv8 [37]	95.2	-	62.1
RGB	Faster R-CNN	88.8	45.7	47.5
	SSD	82.6	31.8	39.8
	YOLOv3	85.9	37.9	43.4
	YOLOv5	90.8	51.9	50.0
	YOLOv8	91.9	-	54.0
RGB+IR	Halfway Fusion [38]	91.4	60.1	55.1
	GAFF	94.0	60.2	55.8
	CFT	97.5	72.9	63.6
	ProbEN	93.4	50.2	51.5
	CSSA	94.3	66.6	59.2
	DIVFusion [39]	89.8	59.9	52.0
	CCFINet	**97.6**	72.6	64.1
	ICAFusion [40]	96.9	71.5	62.2
	CrossFormer	97.4	**76.3**	**66.1**
	AMFusion [41]	96.5	70.1	60.3
	MambaDFuse [42]	95.6	66.4	59.3
	Ours	**97.9**	**76.5**	**66.3**

Note: Methods without citations are already introduced in the introduction or related work sections. Methods with citations are referenced here to provide proper attribution to the original work.

**Table 2:** Comparison of performances on OGSOD datasets measured. Bold red indicates the best; bold blue indicates the second best.

Modality	Method	Year	Oil Tank	Bridge	Harbor	mAP50	mAP
SAR	RetinaNet	2017	17.3	73.3	95.3	62.0	36.7
	YOLOv3	2018	32.4	76.0	97.0	68.5	39.5
	ATSS [43]	2019	26.2	78.0	96.3	66.8	38.6
	YOLOv5	2020	57.7	87.2	97.9	80.9	46.3
	RepPoints [44]	2020	30.4	70.8	95.4	65.5	37.9
	Generalized Focal [45]	2020	33.4	72.8	96.5	67.6	41.8
	Sparse R-CNN [46]	2021	28.7	73.8	94.2	65.6	38.7
	Object BOX [47]	2022	51.0	82.4	96.5	76.6	40.1
	YOLOv7 [48]	2022	59.7	79.8	98.1	79.2	45.1
RGB+SAR	KD	2020	60.3	88.4	98.8	82.6	48.4
	GI Imitation	2021	69.2	**92.9**	99.1	87.1	55.9
	DKD	2022	62.5	62.5	98.7	83.4	49.8
	LD	2022	65.7	65.7	98.3	84.5	51.9
	CoLD	2023	**69.8**	69.8	**99.5**	**87.6**	**56.7**
	Ours	2025	**94.9**	**99.3**	**99.7**	**94.5**	**58.6**

Note: Methods without citations are already introduced in the introduction or related work sections. Methods with citations are referenced here to provide proper attribution to the original work.

**Table 3:** Comparison of performances on Drone Vehicle datasets measured. Bold red indicates the best; bold blue indicates the second best.

Modality	Method	Car	Truck	Freight Car	Bus	Van	mAP0.5	mAP
IR	YOLOv5	90.0	59.5	60.8	89.5	53.8	70.7	-
	YOLOV8	87.96	54.55	17.05	86.16	24.90	54.12	-
	S^2^A-Net [49]	89.7	51.0	50.2	89.0	44.0	64.8	67.5
	Faster R-CNN	89.4	53.5	48.3	87.0	42.6	64.2	-
	ROI Transformer [50]	89.6	51.0	53.4	88.9	44.5	65.5	**70.3**
	Oriented R-CNN [51]	89.6	53.9	53.9	89.2	41.0	65.5	67.0
RGB	YOLOv5	78.6	55.3	43.8	87.1	46.0	-	62.1
	YOLOv8	70.12	54.55	18.2	82.05	22.38	48.57	-
	S^2^A-Net	79.9	50.0	36.2	82.8	37.5	57.3	61.0
	Faster R-CNN	79.0	49.0	37.2	77.0	37.0	-	55.9
	ROI Transformer	61.6	55.1	42.2	85.5	44.8	61.6	61.6
	Oriented R-CNN	80.3	55.4	42.1	86.8	46.9	62.3	60.8
RGB+IR	CFT	98.5	75.0	68.5	82.3	97.3	84.3	61.9
	DDCI	91.0	**78.9**	66.1	90.7	65.5	78.4	-
	CMADet	98.2	70.4	66.4	78.3	96.8	82.0	59.5
	DaFF [52]	92.2	58.9	58.2	71.9	94.4	75.1	45.5
	YOLOFIV	95.89	64.23	34.57	**91.56**	37.29	64.71	-
	C^2^Former	90.2	68.3	64.4	89.8	58.5	74.2	70.0
	ICAFusion	96.1	46.4	34.0	57.1	92.2	65.1	44.0
	COMO [53]	**98.6**	**78.9**	**71.5**	84.1	**97.4**	**86.1**	65.5
	UA-CMDet	88.6	73.0	56.0	88.3	54.8	72.2	64.0
	GM-DETR [54]	92.4	75.3	64.9	80.8	90.8	80.8	55.9
	Ours	**98.7**	**83.1**	**75.8**	**97.3**	**97.6**	**86.3**	**71.7**

Note: Methods without citations are already introduced in the introduction or related work sections. Methods with citations are referenced here to provide proper attribution to the original work.

**Table 4:** Model size, computation cost, and detection speed statistics for different models on the Drone Vehicle dataset.

Methods	Params (M)	Flops@640 (G)	FPS (Hz)
CFT	44.76	17.92	91.74
SuperYOLO	**4.83**	17.98	89.4
GHOST	7.06	20.36	125.6
MFPT	47.65	34.55	51.2
ICAFusion	20.15	14.93	217.4
GM-DETR	70.00	176.00	45.6
DaFF	45.42	18.45	85.2
CMADet	33.33	16.86	208.3
Ours	68.43	**14.36**	**226.2**

**Table 5:** Comparisons of performances with different datasets in terms of mAP50, mAP75, and mAP.

Dataset	Modality	Method	mAP50	mAP75	mAP
LLVIP	IR	Baseline	94.6	72.2	61.9
	RGB		90.8	51.9	50.0
	RGB+IR	+ Two Stream	95.8	71.4	62.3
		+ MSFEM	96.3	73.5	63.5
		+ MSFEM + FIM	97.1	75.3	64.7
		+ MSFEM + FIM + FCFM	**97.9 (3.3⬆)**	**76.5 (4.3⬆)**	**66.3 (4.4⬆)**
OGSOD	RGB	Baseline	80.9	50.31	46.3
	SAR		78.7	35.5	40.2
	RGB+SAR	+ Two Stream	81.4	58.3	45.63
		+ MSFEM	86.2	65.0	49.78
		+ MSFEM + FIM	88.9	68.2	53.4
		+ MSFEM + FIM + FCFM	**94.5 (13.6⬆)**	**74.3 (23.99⬆)**	**58.6 (12.3⬆)**
Drone Vehicle	IR	Baseline	80.8	58.7	60.2
	RGB		74.6	46.9	46.7
	RGB+IR	+ Two Stream	81.3	64.6	63.4
		+ MSFEM	84.1	69.58	68.3
		+ MSFEM + FIM	85.3	72.48	69.7
		+ MSFEM + FIM + FCFM	**86.3 (5.5⬆)**	**76.4 (17.7⬆)**	**71.7 (8.1⬆)**

**Table 6:** Ablation study of spatial vs. channel correction sequence.

FCFM	mAP50	mAP75	mAP
Channel → Spatial	96.4	73.3	63.5
Spatial → Channel	97.9	76.5	66.3

## Data Availability

The LLVIP dataset (https://drive.google.com/file/d/1VTlT3Y7e1h-Zsne4zahjx5q0TK2ClMVv/view, accessed on 10 August 2025), OGSOD dataset (OGSOD-1.0.zip—Google Drive), and Drone Vehicle dataset (VisDrone/DroneVehicle: Drone-based RGB-Infrared Cross-Modality Vehicle Detection via Uncertainty-Aware Learning (github.com, accessed on 10 Auust 2025)) were utilized.
